# Supplementary material for: Targeting TRIP13 in favorable histology Wilms tumor with nuclear export inhibitors synergizes with doxorubicin
Source: Commun Biol. 2024 Apr 8;7:426. doi: 10.1038/s42003-024-06140-6 (PMC11001930; doi:10.1038/s42003-024-06140-6)
Supplement: Supplementary file 2 — Supplementary Information [file 42003_2024_6140_MOESM2_ESM.pdf]

**Supplementary Figure 1: Correlation between shRNA and sgRNA loss of function screens among biological replicates.**

RNAi screens by cell lines represented in (a). Correlations of RNAi screens in (b) PEDS_0002T, (c) PEDS_0041_T1, (d) PEDS_0041_T2. CRISPR-Cas9 screens used early and late passages to determine change in abundance of sgRNAs summarized by cell lines in (e). as shown in f-k. (f) PEDS_0002T early passage, (g) PEDS_0002T late passage, (h) PEDS_0023_T1 early passage, (i) PEDS_0023_T1 late passage, (j) PEDS_0023_T2 early passage, (k) PEDS_0023_T2 late passage. Right color gradient delineates the Pearson's correlation with anticorrelation as red and correlation as blue.

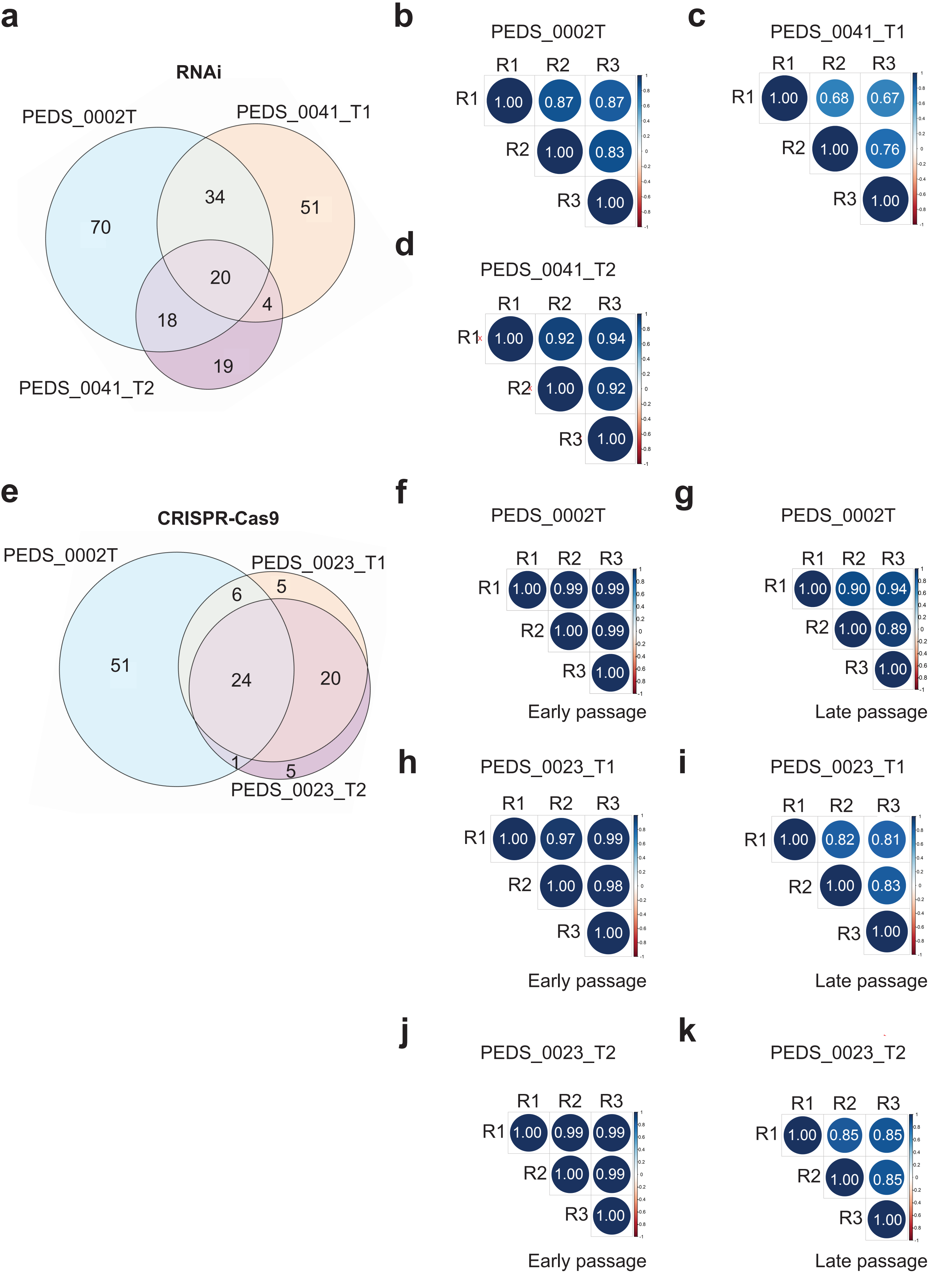
a
RNAi
PEDS_0002T
PEDS_0041_T1
PEDS_0041_T2
70
34
51
20
18
4
19
b
PEDS_0002T
R1 R2 R3
R1 1.00 0.87 0.87
R2 1.00 0.83
R3 1.00
c
PEDS_0041_T1
R1 R2 R3
R1 1.00 0.68 0.67
R2 1.00 0.76
R3 1.00
d
PEDS_0041_T2
R1 R2 R3
R1 1.00 0.92 0.94
R2 1.00 0.92
R3 1.00
e
CRISPR-Cas9
PEDS_0002T
PEDS_0023_T1
PEDS_0023_T2
51
6
5
24
20
1
5
f
PEDS_0002T
R1 R2 R3
R1 1.00 0.99 0.99
R2 1.00 0.99
R3 1.00
Early passage
g
PEDS_0002T
R1 R2 R3
R1 1.00 0.90 0.94
R2 1.00 0.89
R3 1.00
Late passage
h
PEDS_0023_T1
R1 R2 R3
R1 1.00 0.97 0.99
R2 1.00 0.98
R3 1.00
Early passage
i
PEDS_0023_T1
R1 R2 R3
R1 1.00 0.82 0.81
R2 1.00 0.83
R3 1.00
Late passage
j
PEDS_0023_T2
R1 R2 R3
R1 1.00 0.99 0.99
R2 1.00 0.99
R3 1.00
Early passage
k
PEDS_0023_T2
R1 R2 R3
R1 1.00 0.85 0.85
R2 1.00 0.85
R3 1.00
Late passage

**Supplementary Figure 2: Inhibition of nuclear export in FHWT by KPT-330 or sh*XPO1*.**

(a) Suppression of XPO1 by immunoblot (b) Change in viability using sh*XPO1* across FHWT cell lines as compared to shControl. * p-value <0.05, ** p-value <0.005, *** p-value <0.0005. Error bars represent mean ± SD. (c) Additional replicate of the KPT-330 treated (5μM treat for 48 hours) Wilms tumor cells across 5 different cell lines.

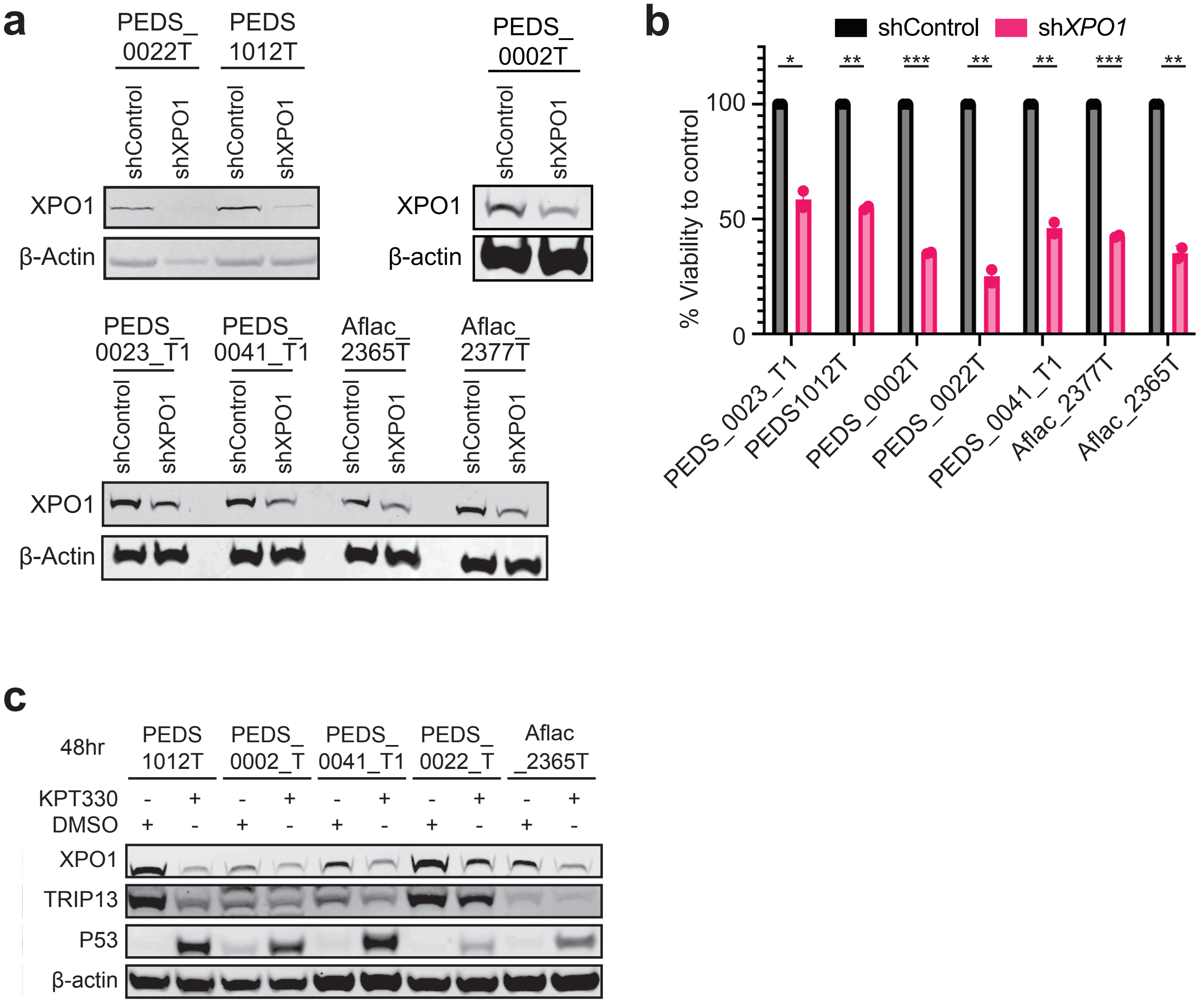
a
PEDS_0022T
PEDS 1012T
shControl
shXPO1
shControl
shXPO1
XPO1
β-Actin
PEDS_0002T
shControl
shXPO1
XPO1
β-actin
PEDS_0023_T1
PEDS_0041_T1
Aflac_2365T
Aflac_2377T
shControl
shXPO1
XPO1
β-Actin
b
shControl
shXPO1
% Viability to control
0
50
100
*
**
***
**
**
***
**
PEDS_0023_T1
PEDS1012T
PEDS_0002T
PEDS_0022T
PEDS_0041_T1
Aflac_2377T
Aflac_2365T
c
48hr
PEDS 1012T
PEDS_0002_T
PEDS_0041_T1
PEDS_0022_T
Aflac_2365T
KPT330
DMSO
XPO1
TRIP13
P53
β-actin

**Supplementary Figure 3: Dose response curves to KPT-330 across cells lines**

KPT-330 IC50 data from all cell lines depicted in **Fig 3d**. Matched tumor normal pairs are plotted together for three of the cell lines. Error bars represent mean ± SD from two biological replicates.

KPT-330 IC50 Curves

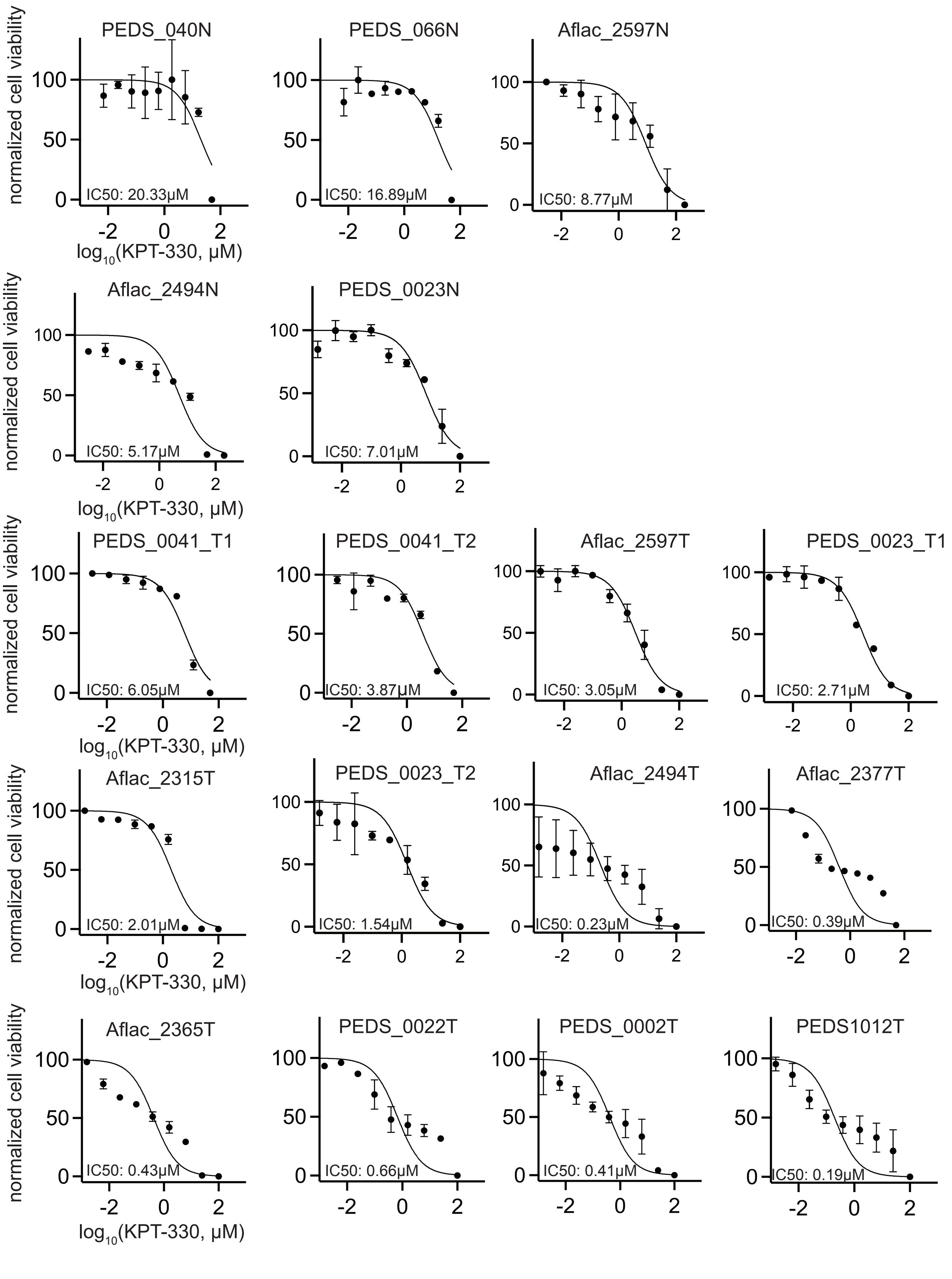

**Supplementary Figure 4: Response to KPT-330 Treatment.**

(a) Plot measuring correlation between XPO1 expression levels and corresponding KPT-330 IC50. (b) qRT-PCR analysis of *XPO1* in KPT-330 and DMSO treated cells. Data was normalized by the amount of TBP expressed relative to the corresponding value for all the cells and are means ± SD from at least two biological replicates. * p-value<0.05, all comparisons represent a Student's unpaired two-sided t-test. (c) Cell lines were treated with 5μM of KPT-330 for 24 hours, XPO1/CRM levels are suppressed with accumulation of p53 across a majority of cell lines with exception to PEDS_0023_T1 which harbors a TP53 mutation. (d) Normal cell lines were treated with 5μM of KPT-330 for 24 hours and accumulation of p53 was assessed. (e) Immunoblots from Supp Fig 4c and 4d were quantified to determine the accumulation of p53 relative to β-actin, excluding PEDS_0023_T1. ** p-value<0.005, all comparisons represent a Student's t-test.

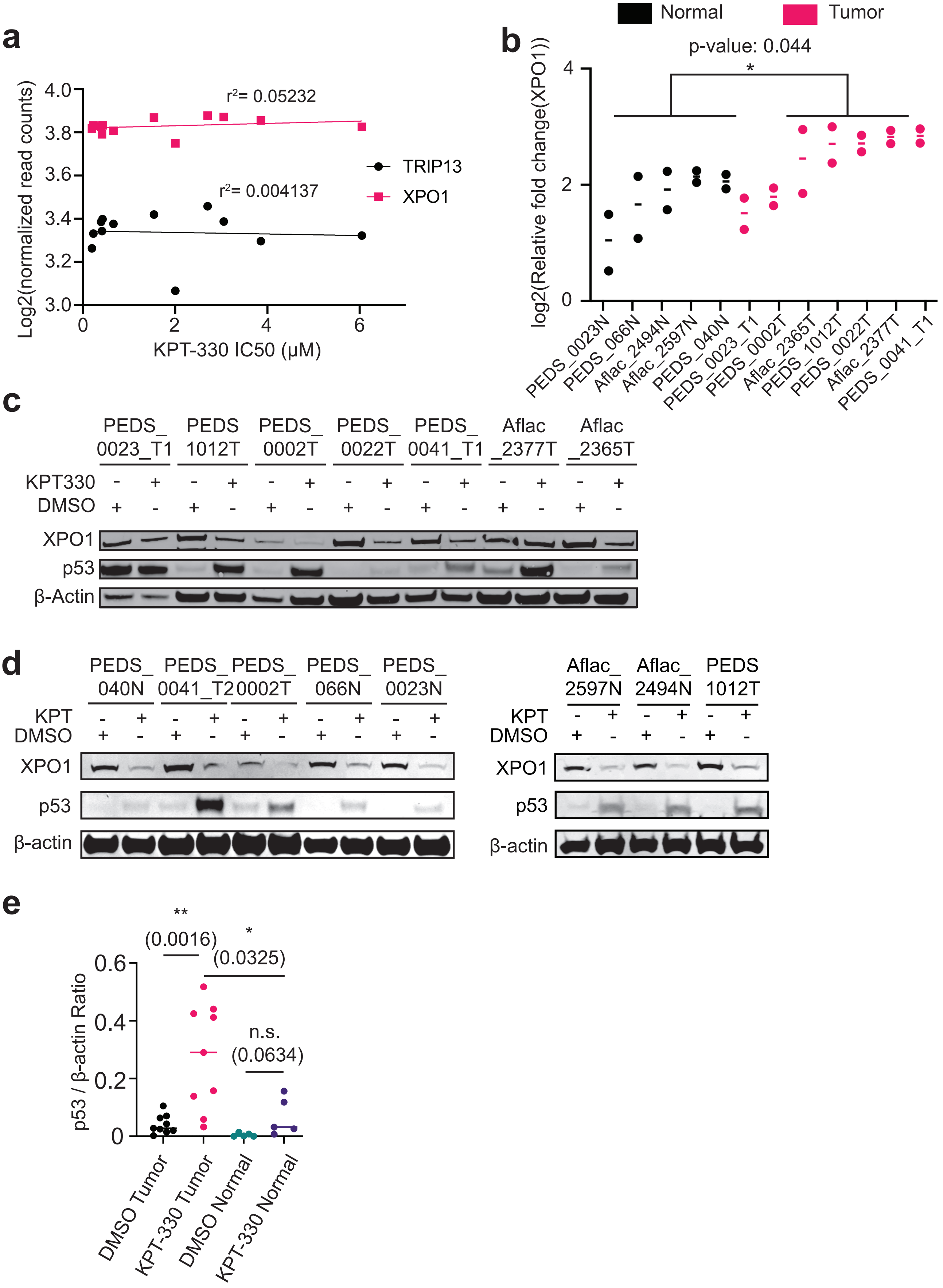

a
Log2(normalized read counts)
4.0
3.8
3.6
3.4
3.2
3.0
0
2
4
6
KPT-330 IC50 (μM)
r²= 0.05232
r²= 0.004137
TRIP13
XPO1
b
Normal
Tumor
p-value: 0.044
*
log2(Relative fold change(XPO1))
4
2
0
PEDS_0023N
PEDS_066N
Aflac_2494N
Aflac_2597N
PEDS_040N
PEDS_0023_T1
PEDS_0002T
Aflac_2365T
PEDS_1012T
PEDS_0022T
Aflac_2377T
PEDS_0041_T1
c
PEDS_ 0023_T1
PEDS 1012T
PEDS_ 0002T
PEDS_ 0022T
PEDS_ 0041_T1
Aflac _2377T
Aflac _2365T
KPT330
DMSO
XPO1
p53
β-Actin
d
PEDS_ 040N
PEDS_ 0041_T2
PEDS_ 0002T
PEDS_ 066N
PEDS_ 0023N
Aflac_ 2597N
Aflac_ 2494N
PEDS 1012T
KPT
DMSO
XPO1
p53
β-actin
e
p53 / β-actin Ratio
0.6
0.4
0.2
0
**
(0.0016)
*
(0.0325)
n.s.
(0.0634)
DMSO Tumor
KPT-330 Tumor
DMSO Normal
KPT-330 Normal

**Supplementary Figure 5: Inhibition of nuclear export and TRIP13 in FHWT cell lines.** (a) Immunoblots depicting the decrease in protein levels of XPO1 in the cytoplasmic lysates and nuclear accumulation of p53 in the nuclear lysates upon treatment with KPT-330 at 24 hr. (b) We introduced sgRNAs targeting either LacZ or TP53 and confirmed increase in p53 following treatment with KPT-330 in the LacZ controls. (c) Dose-response curves for the sgTP53 and sgLacZ cells for KPT-330 in PEDS_0022T. Error bars represent mean ± SD and represent biological replicates. (d) Dot plots representing the expression levels of TRIP13 in Wilms tumor when compared with the normal matched kidney tissue. Immunoblot depicting suppression of TRIP13 in (e) CCLF_PEDS1012T (f) CCLF_PEDS_0022T, and (g) Aflac_2377T. (h) Overexpression of TRIP13 leads to modest increase in proliferation as compared to luciferase. (i) p53 and p21 protein levels increase while TRIP13 levels decrease upon KPT-330 treatment in TP53 wildtype cells.

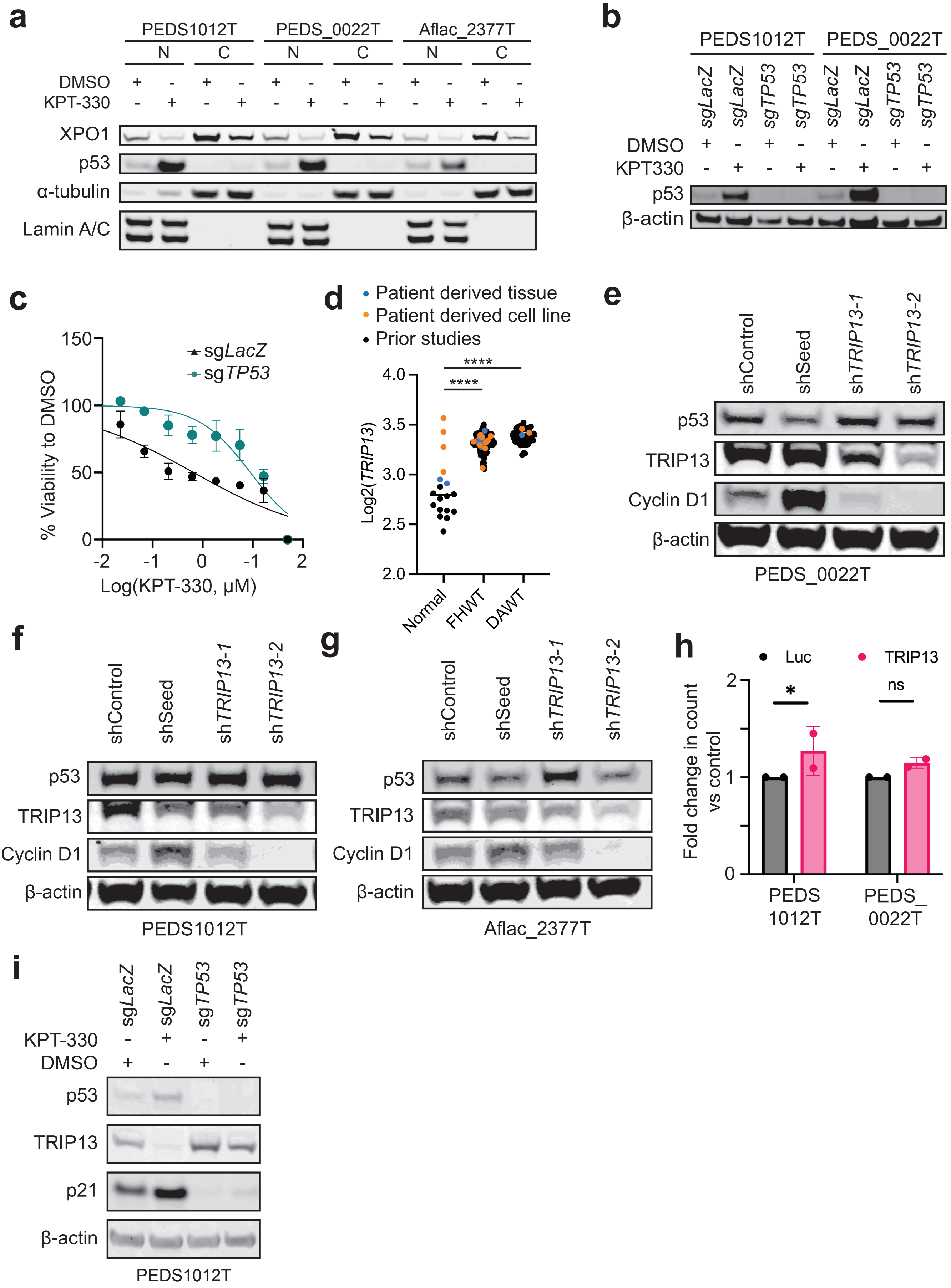

a
PEDS1012T
PEDS_0022T
Aflac_2377T
N
C
DMSO
KPT-330
XPO1
p53
α-tubulin
Lamin A/C
b
PEDS1012T
PEDS_0022T
sgLacZ
sgTP53
DMSO
KPT330
p53
β-actin
c
% Viability to DMSO
sgLacZ
sgTP53
Log(KPT-330, μM)
d
Patient derived tissue
Patient derived cell line
Prior studies
Log2(TRIP13)
****
Normal
FHWT
DAWT
e
shControl
shSeed
shTRIP13-1
shTRIP13-2
p53
TRIP13
Cyclin D1
β-actin
PEDS_0022T
f
shControl
shSeed
shTRIP13-1
shTRIP13-2
p53
TRIP13
Cyclin D1
β-actin
PEDS1012T
g
shControl
shSeed
shTRIP13-1
shTRIP13-2
p53
TRIP13
Cyclin D1
β-actin
Aflac_2377T
h
Luc
TRIP13
Fold change in count vs control
*
ns
PEDS 1012T
PEDS_ 0022T
i
sgLacZ
sgTP53
KPT-330
DMSO
p53
TRIP13
p21
β-actin
PEDS1012T

**Supplementary Figure 6: Dose response curves to doxorubicin across cells lines**

Wilms tumor response to doxorubicin treatment. Doxorubicin IC50 data from all cell lines depicted in **Fig 5b**. Matched tumor normal pairs are plotted together for three of the cell lines. Error bars represent mean ± SD from two biological replicates.

Doxorubicin IC50 Curves

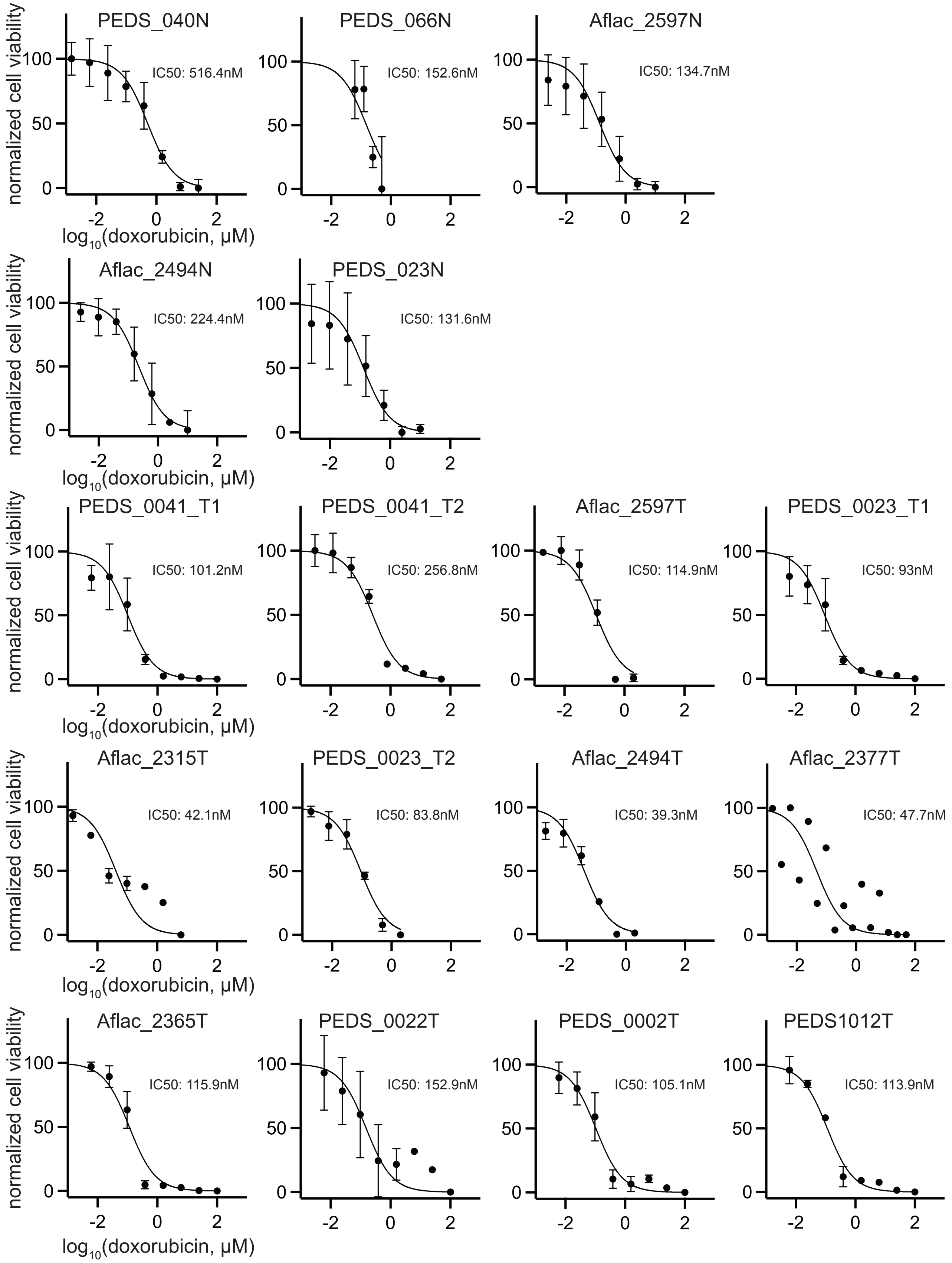

## Supplementary Figure 7: Gating Strategy for Flow Cytometry

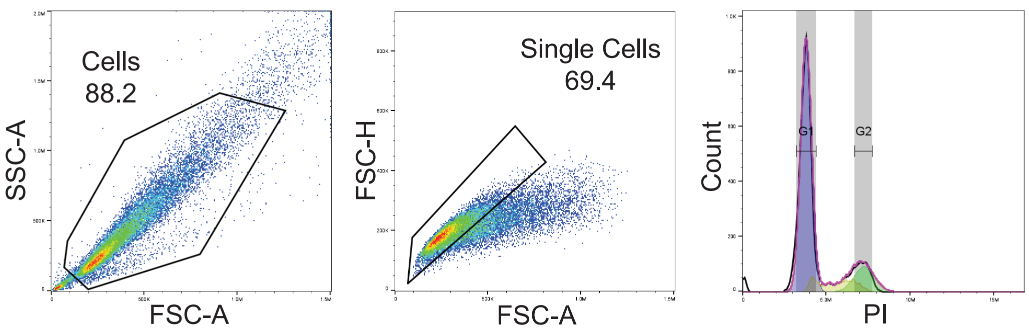


**RMSD**: 4.89
**%G1**: 73.4
**%S**: 12.3
**%G2**: 14.3
**G1 Mean**: 3.77E6
**G2 Mean**: 7.26E6
**G1 CV**: 11.9
**G2 CV**: 11.9
**% less G1**: 4.10
**% greater G2**: -0.93

**Supplementary Figure 8: The full, unedited blots in Figures.**

# Fig 3e

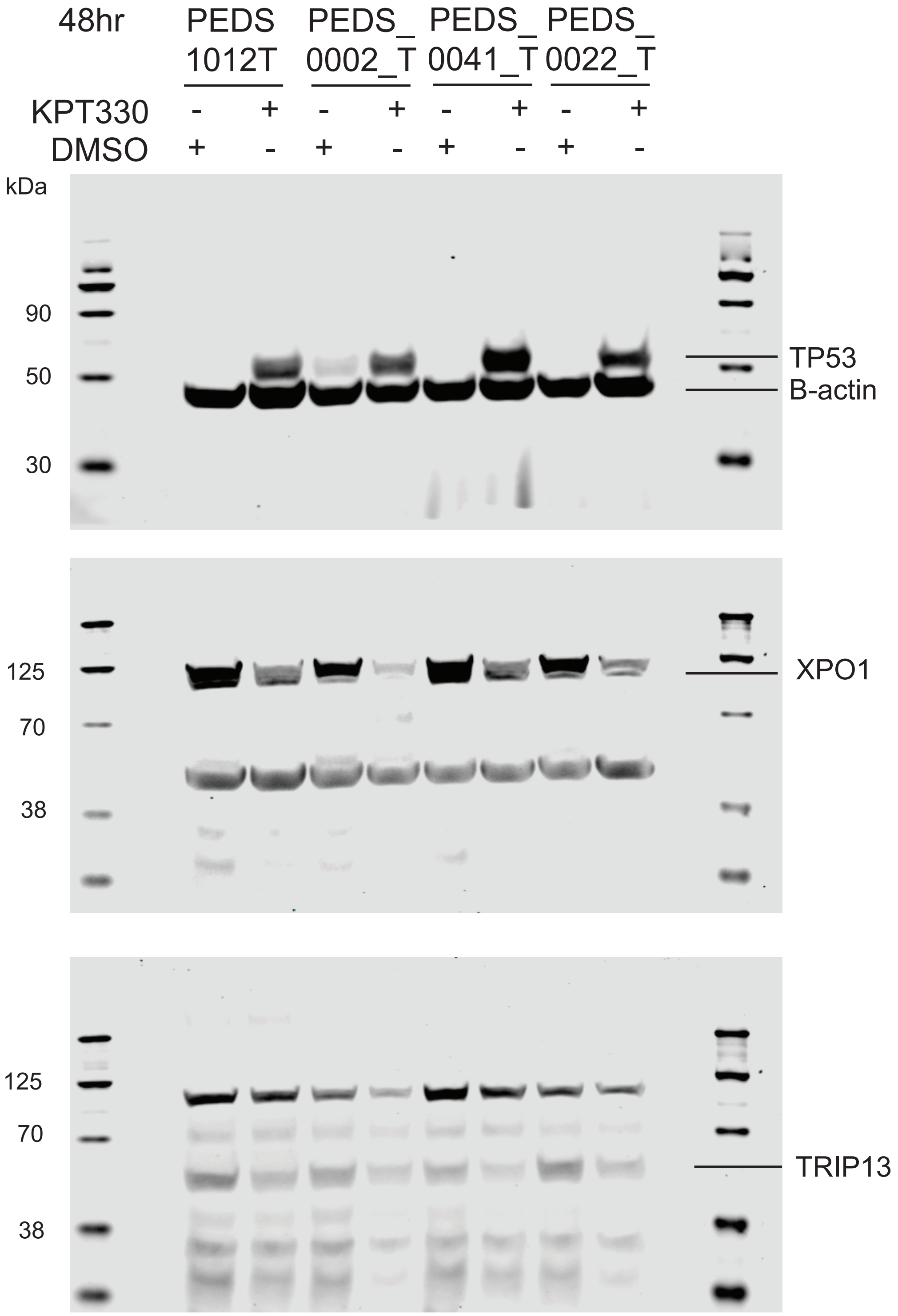
48hr
PEDS 1012T
PEDS_ 0002_T
PEDS_ 0041_T
PEDS_ 0022_T
KPT330 - + - + - + - +
DMSO + - + - + - + -
kDa
90
50
30
TP53
B-actin
125
70
38
XPO1
125
70
38
TRIP13

# Fig S2A

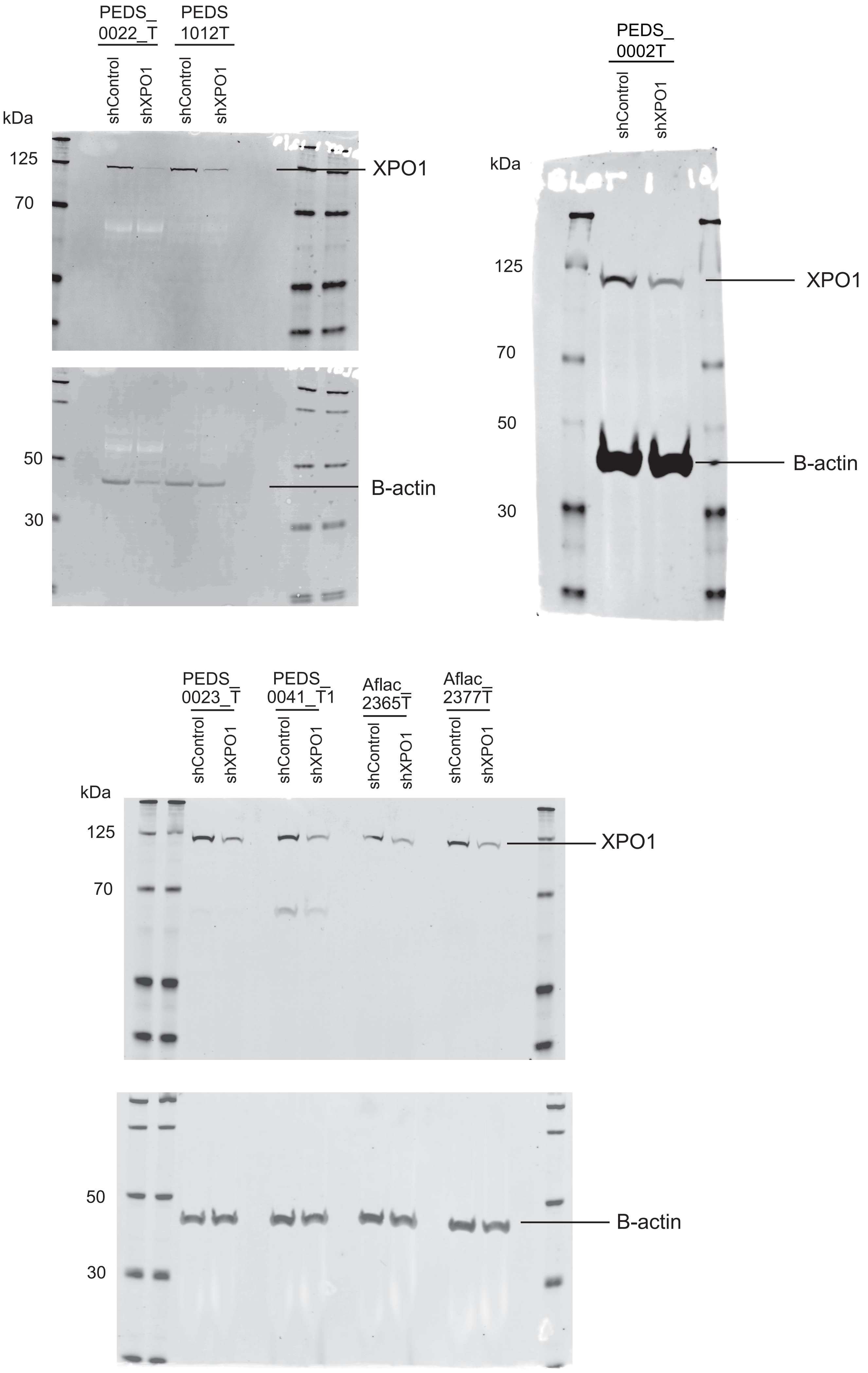

PEDS_
0022_T
PEDS
1012T
shControl
shXPO1
shControl
shXPO1
kDa
125
70
XPO1
50
30
B-actin
PEDS_
0002T
shControl
shXPO1
kDa
125
70
50
30
XPO1
B-actin
PEDS_
0023_T
PEDS_
0041_T1
Aflac_
2365T
Aflac_
2377T
shControl
shXPO1
shControl
shXPO1
shControl
shXPO1
shControl
shXPO1
kDa
125
70
XPO1
50
30
B-actin

Fig S2C

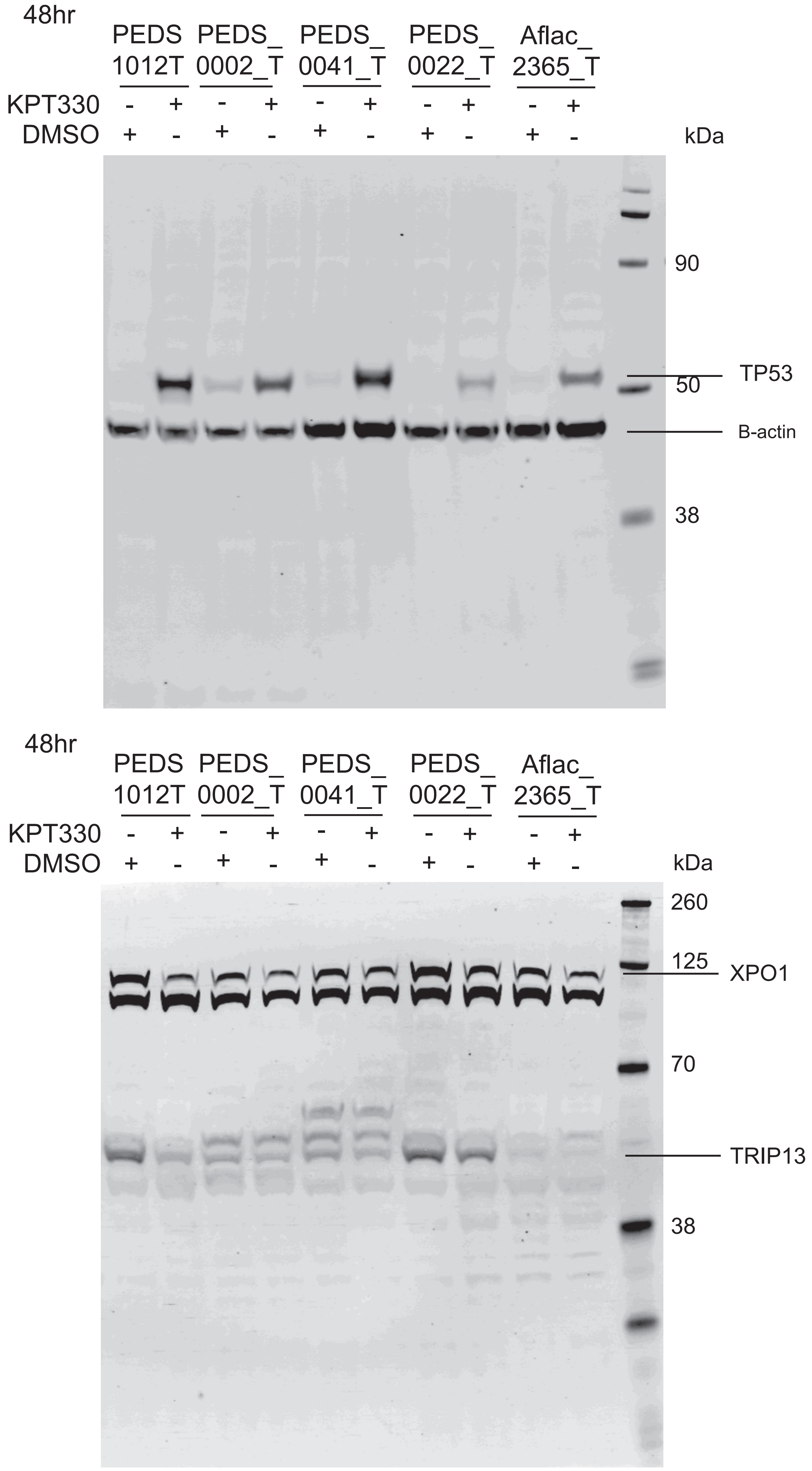
48hr
PEDS 1012T
PEDS_ 0002_T
PEDS_ 0041_T
PEDS_ 0022_T
Aflac_ 2365_T
KPT330 - + - + - + - + - +
DMSO + - + - + - + - + -
kDa
90
50
38
TP53
B-actin
48hr
PEDS 1012T
PEDS_ 0002_T
PEDS_ 0041_T
PEDS_ 0022_T
Aflac_ 2365_T
KPT330 - + - + - + - + - +
DMSO + - + - + - + - + -
kDa
260
125
70
38
XPO1
TRIP13

Fig S4C

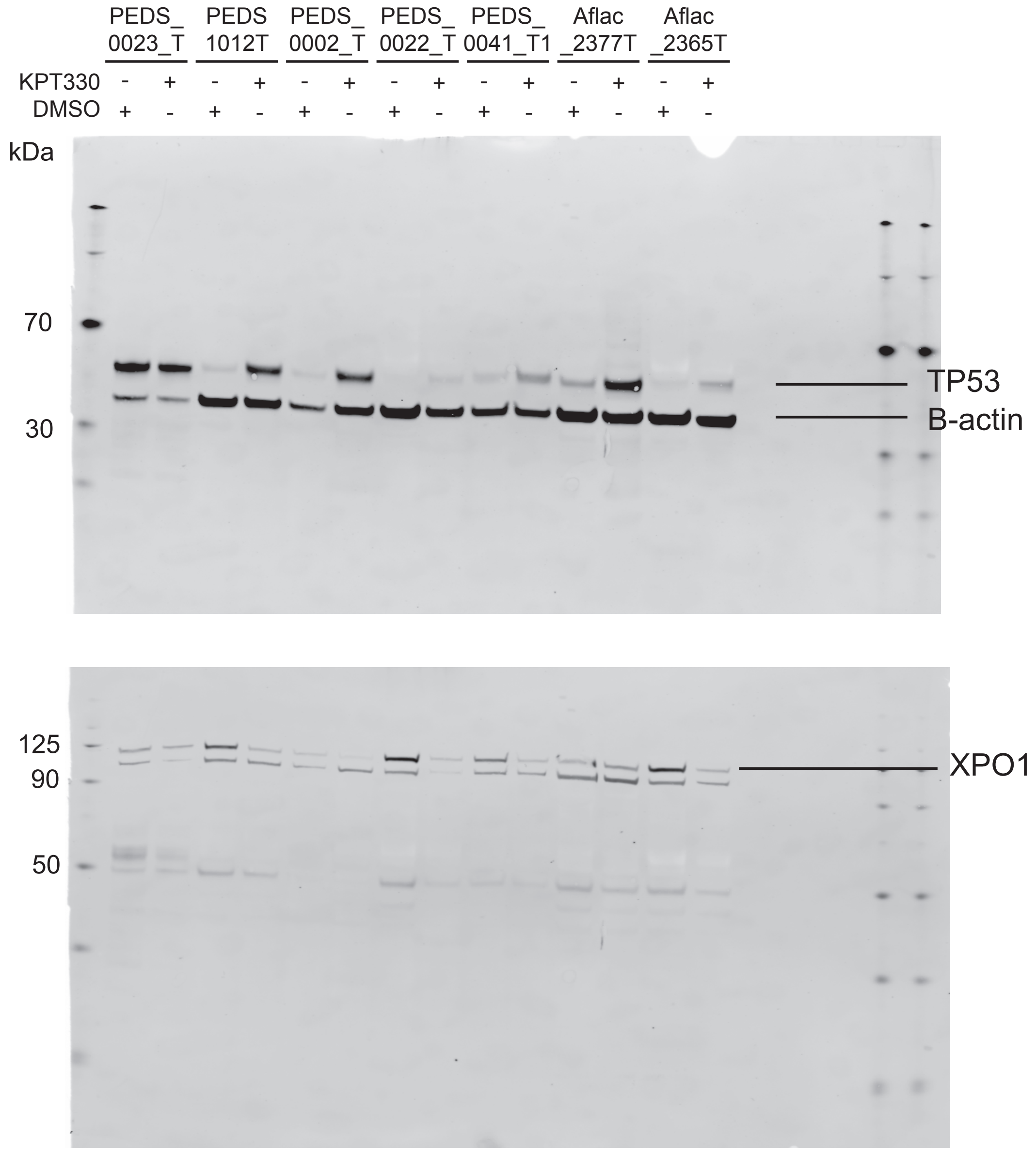


Fig S4D

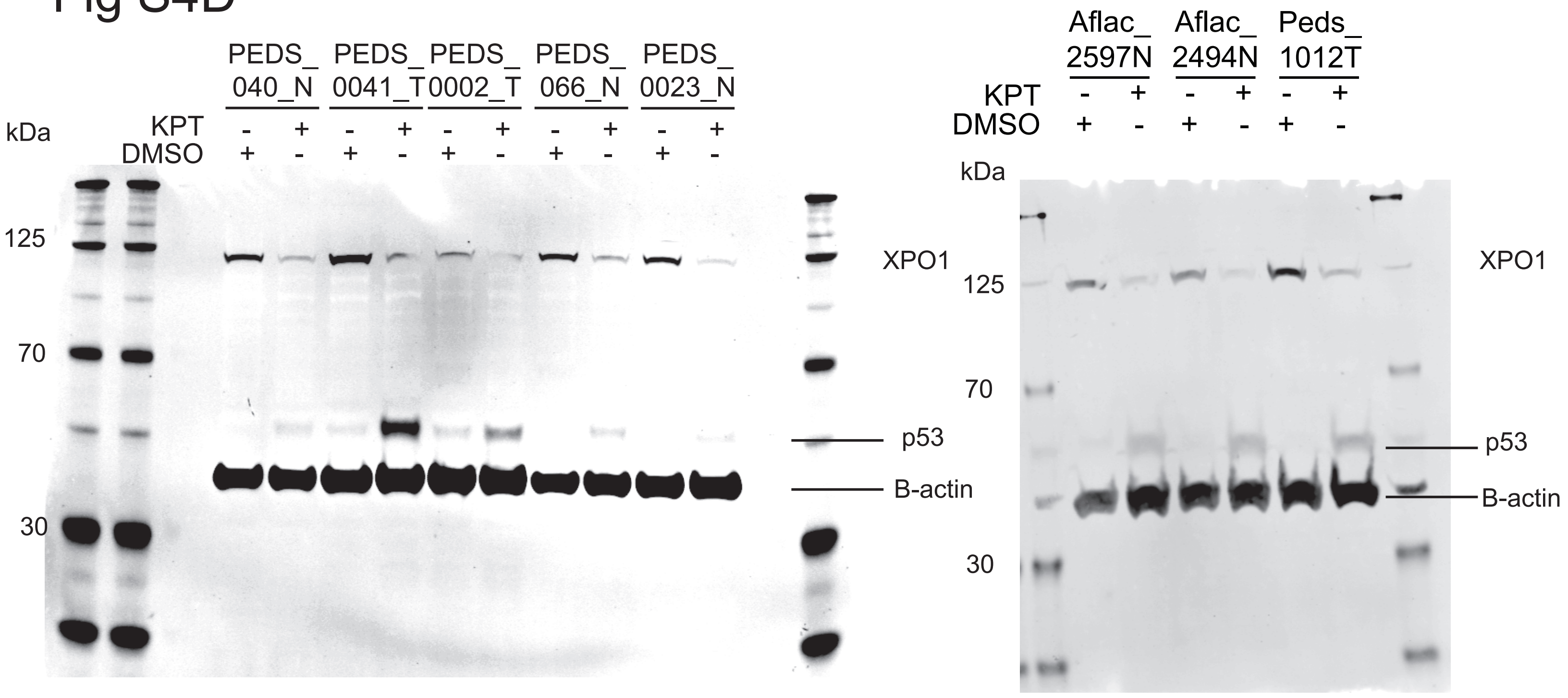

# Fig S5A

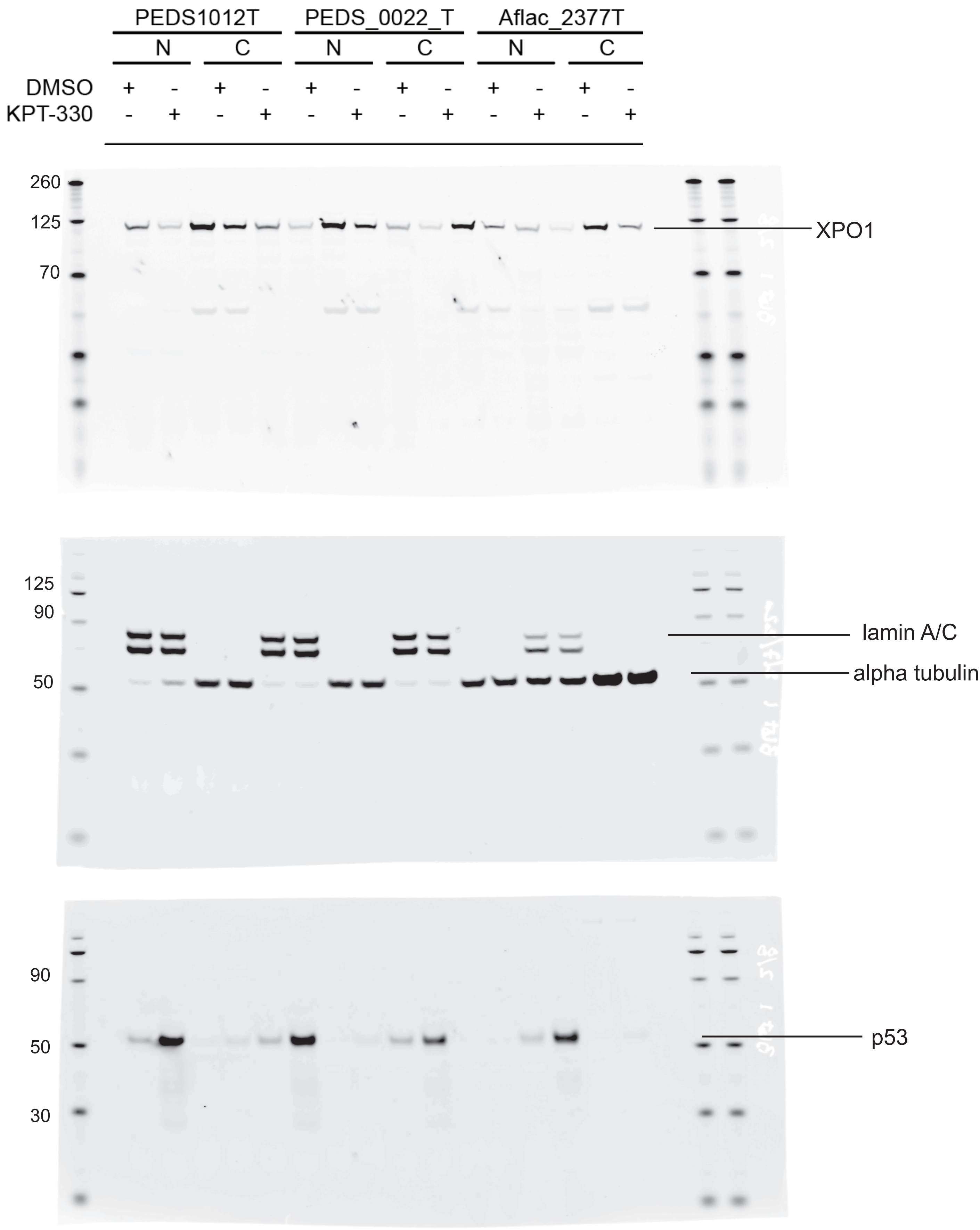

PEDS1012T
PEDS_0022_T
Aflac_2377T
N
C
N
C
N
C
DMSO + - + - + - + - + - + -
KPT-330 - + - + - + - + - + - +
260
125
70
XPO1
125
90
50
lamin A/C
alpha tubulin
90
50
30
p53

Fig S5b

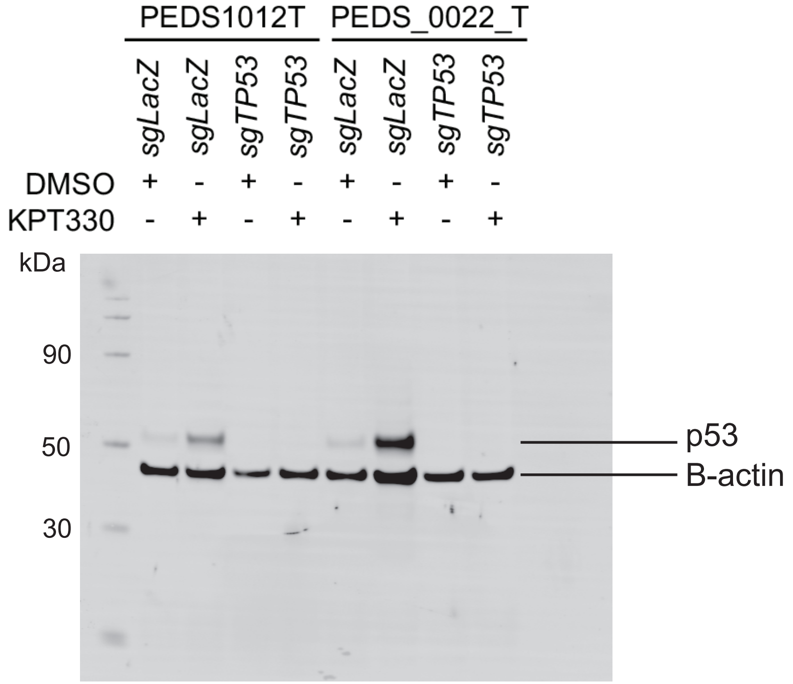


Fig S5e-g

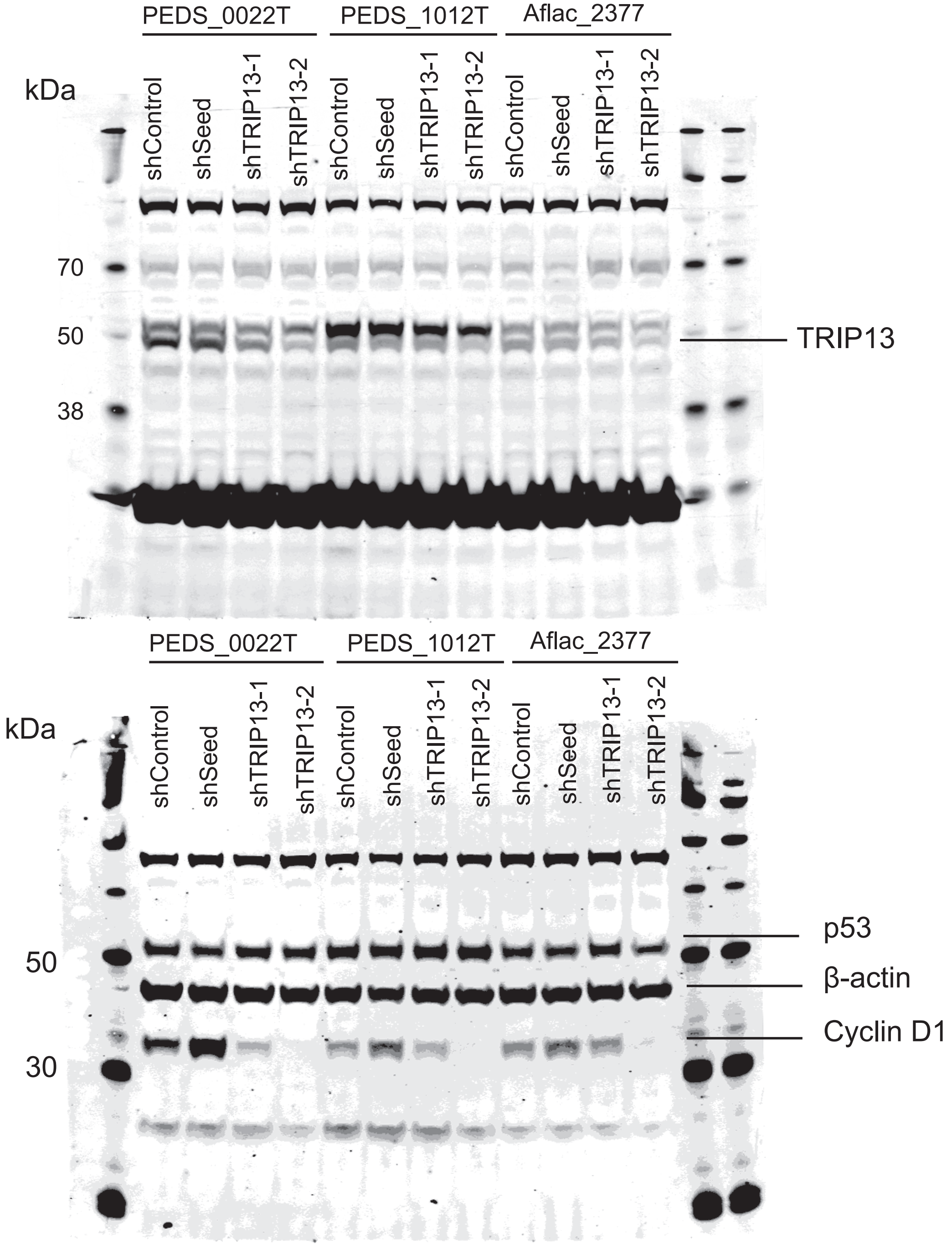

# Fig S5I

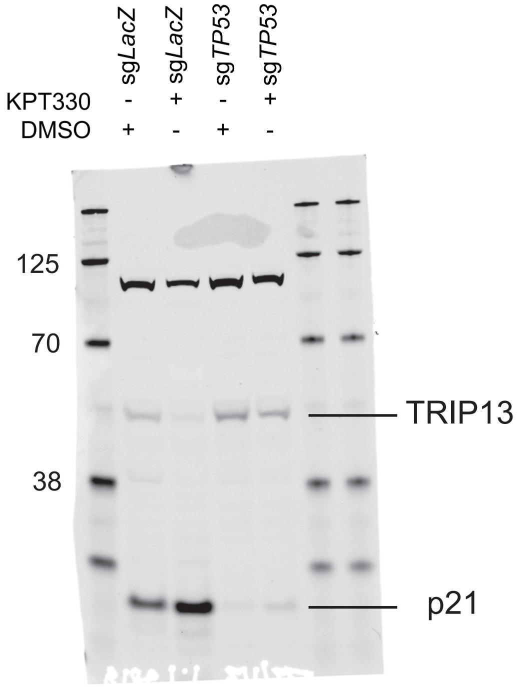
sgLacZ
sgLacZ
sgTP53
sgTP53
KPT330 - + - +
DMSO + - + -
125
70
38
TRIP13
p21

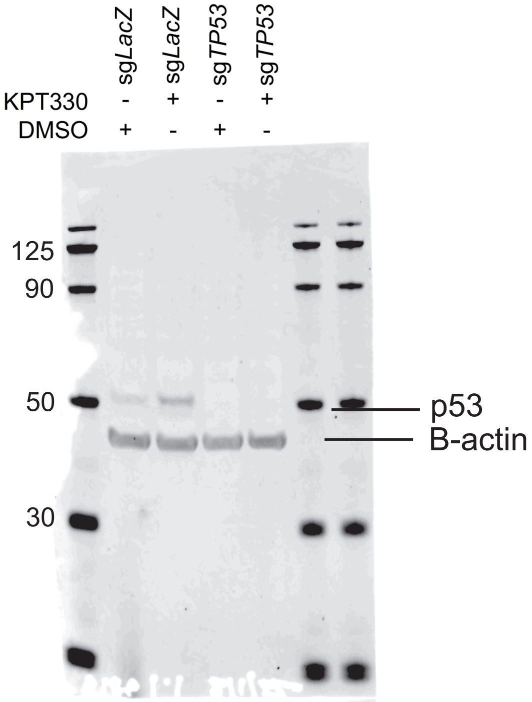
sgLacZ
sgLacZ
sgTP53
sgTP53
KPT330 - + - +
DMSO + - + -
125
90
50
30
p53
B-actin

**Supplementary Table 1:F-Media Components**

| | |
|---|---|
| DMEM | 500 mL |
| DMEM:F12 | 500 mL |
| Hydrocortisone/EGF mix | 1.0 mL |
| Insulin (5mg/mL) | 1.0 mL |
| FBS | 50 mL |
| Glutamine | 10 mL |
| Pen/Strep | 5 mL |
| Cholera Toxin (11.7uM) | 8.6 uL |
| Y-27632 (ROCK inhibitor) | 0.5 mL |

**Stock Solutions:**

| | |
|---|---|
| Hydrocortisone/EGF Mix (Sigma # H-0888) | Dissolve hydrocortisone in 100% ethanol to 0.5 ug/mL. Mix 1 mL of this with 19 mL DMEM containing 2.5 ug EGF |
| Insulin (Sigma # I-550) | Dissolve 100 mg insulin in 20 mL of distilled water contaiing 200 uL glacial acetic acid. |
| Cholera Toxin (Sigma # C-8052) | Dissolve 1 mg of cholera toxin in 1 mL of distilled water |
| Y-27632 (Selleck S1049) | Dissolve to stock concentration of 10 mM |

**Supplementary Table 2: Cell Line Passage Information**

| Cell Line | Passages before senesc | Media | Detached with | Passage at RNA Sequencing |
|---|---|---|---|---|
| Aflac_2315_T | 14-15 | F-media | TrypLE | 6 |
| Aflac_2365_T | 12-14 | F-media | TrypLE | 9 |
| Aflac_2377_T | 14-15 | F-media | TrypLE | 4 |
| Aflac_2494_N | 10 | F-media | TrypLE | 3 |
| Aflac_2494_T | 38-40 | F-media | TrypLE | 16 |
| Aflac_2597_N | 10 | F-media | TrypLE | <5 |
| Aflac_2597_T | 9 | F-media | TrypLE | <5 |
| PEDS_0002_T | >50 | F-media | TrypLE | 26 |
| PEDS_0022_N | 10 | F-media | TrypLE | 5 |
| PEDS_0022_T | 30 | F-media | TrypLE | 19 |
| PEDS_0023_T | 28-32 | F-media | TrypLE | 7 |
| PEDS_0023_T2 | 25-30 | F-media | TrypLE | 8 |
| PEDS_0041_T1 | 14-15 | F-media | TrypLE | 7 |
| PEDS_0041_T2 | 10 | F-media | TrypLE | 5 |
| PEDS1012T | 40-45 | F-media | TrypLE | 24 |

**Supplementary Table 3: FACS data**

| **PEDS_1012T** | G1 | | | S | | | G2 | | | < G1 | | | > G2 | | |
|---|---|---|---|---|---|---|---|---|---|---|---|---|---|---|---|
| DMSO | 57.6 | 60.9 | 60.8 | 17.5 | 17.6 | 10 | 17.7 | 16.5 | 19.7 | 7.26 | 3.52 | 3.75 | 0.19 | 0.049 | 0.71 |
| KPT330 | 42.5 | 47.7 | 49.4 | 8.34 | 7.68 | 6.84 | 41.9 | 35.5 | 37.7 | 6.28 | 5.1 | 1.73 | 0.42 | 0.29 | 2.25 |

| **PEDS_0022_T** | G1 | | | S | | | G2 | | | < G1 | | | > G2 | | |
|---|---|---|---|---|---|---|---|---|---|---|---|---|---|---|---|
| DMSO | 67.3 | 40.7 | 64.1 | 10.9 | 26.3 | 13 | 10.2 | 13 | 13.9 | 3.97 | 13.4 | 7.31 | 0.61 | 0.85 | 0.51 |
| KPT330 | 54.5 | 70.4 | 50 | 3.84 | 2.17 | 4.29 | 35.6 | 25.2 | 32.2 | 1.52 | 2.2 | 7.06 | 0.19 | 1.62 | 0.94 |

| **Aflac_2377T** | G1 | | S | | G2 | | <G1 | | >G2 | |
|---|---|---|---|---|---|---|---|---|---|---|
| DMSO | 52.5 | 55.7 | 18 | 16.8 | 13.6 | 14.5 | 9.47 | 8.28 | 1.48 | 1.86 |
| KPT330 | 52.9 | 61.6 | 7.83 | 5.86 | 24 | 21.3 | 10.7 | 8.86 | 0.53 | 0.76 |

**Supp Table 4 - Primer Sequences**

| Name | Sequence | Type |
|---|---|---|
| shTRIP13-1 Fwd | CCGGCGATTATGTGATGACAACTTTCTCGAGAAAGTTGTCATCACATAATCGTTTTTG | shRNA |
| shTRIP13-1 Rev | AATTCAAAAACGATTATGTGATGACAACTTTCTCGAGAAAGTTGTCATCACATAATCG | shRNA |
| shTRIP13-2 Fwd | CCGGGCACTGTTGCACTTCACATTTCTCGAGAAATGTGAAGTGCAACAGTGCTTTTTG | shRNA |
| shTRIP13-2 Rev | AATTCAAAAAGCACTGTTGCACTTCACATTTCTCGAGAAATGTGAAGTGCAACAGTGC | shRNA |
| shTRIP13-3 Fwd | CCGGCACCTGTAATCCCAGCACTTTCTCGAGAAAGTGCTGGGATTACAGGTGTTTTTG | shRNA - 3'UTR |
| shTRIP13-3 Rev | AATTCAAAAACACCTGTAATCCCAGCACTTTCTCGAGAAAGTGCTGGGATTACAGGTG | shRNA - 3'UTR |
| shTRIP13-2 Ctrl Fwd | CCGGGCACTGTTGTGCTTCACATTTCTCGAGAAATGCAAAGTGCAACAGTGCTTTTTG | shRNA Control |
| shTRIP13-2 Ctrl Rev | AATTCAAAAAGCACTGTTAGTCTTCACATTTCTCGAGAAATGTGAGCAGCAACAGTGC | shRNA Control |
| TRIP13 Fwd | CGTGCTGATTGATGAGGTGG | qRTPCR |
| TRIP13 Rev | ACGTCGATCTTCTCGGTGAT | qRTPCR |
| shXPO1 Ctrl Fwd | CCGGGCTCAAGTTCTACTGACACATCTCGAGGCTCAAGTTCTACTGACACAT TTTTTG | shRNA |
| shXPO1 Ctrl Rev | AATTCAAAAAGCTCAAGTTCTACTGACACATCTCGAGGCTCAAGTTCTACTGACACAT | shRNA |
| shXPO1 Fwd | CCGGGCTCAAGAAGTACTGACACATCTCGAGGCTCAAGAAGTACTGACACAT TTTTTG | shRNA |
| shXPO1 Rev | AATTCAAAAAGCTCAAGAAGTACTGACACATCTCGAGGCTCAAGAAGTACTGACACAT | shRNA |
| TBP Fwd | TTCGGAGAGTTCTGGGATTGTA | qRTPCR housekeeping |
| TBP Rev | TGGACTGTTCTTCACTCTTGGC | qRTPCR housekeeping |
| XPO1 Fwd | GCAGTTGGTTCAATCTCTGGTAAT | qRTPCR |
| XPO1 Rev | AAATCAAGCAGCTGACGAGC | qRTPCR |
