# Supplementary material for: Targeting TRIP13 in favorable histology Wilms tumor with nuclear export inhibitors synergizes with doxorubicin
Source: Commun Biol. 2024 Apr 8;7:426. doi: 10.1038/s42003-024-06140-6 (PMC11001930; doi:10.1038/s42003-024-06140-6)
Supplement: Supplementary file 3 — Description of Additional Supplementary Files [file 42003_2024_6140_MOESM3_ESM.pdf]

Description of Additional Supplementary Files

File name: Supplementary Data 1
Description: Alterations.

File name: Supplementary Data 2
Description: Screen Data.

File name: Supplementary Data 3
Description: PEDS_0041_T1 comparison of KPT330 treated cells vs DMSO control.

File name: Supplementary Data 4
Description: KPTvDMSO GSEA Hallmarks.

File name: Supplementary Data 5
Description: shTRIP13 differentially expressed genes.

File name: Supplementary Data 6
Description: shTRIP13 vs shControl GSEA Hallmarks.

File name: Supplementary Data 7
Description: Differentially Expressed Genes in shTRIP13 and KPT330 Conditions.

File name: Supplementary Data 8
Description: The source data behind the graphs in the paper.
