# Supplementary material for: Targeting TRIP13 in favorable histology Wilms tumor with nuclear export inhibitors synergizes with doxorubicin
Source: Commun Biol. 2024 Apr 8;7:426. doi: 10.1038/s42003-024-06140-6 (PMC11001930; doi:10.1038/s42003-024-06140-6)
Supplement: Supplementary file 6 — Reporting Summary [file 42003_2024_6140_MOESM6_ESM.pdf]

nature portfolio

Corresponding author(s): William C. Hahn, Andrew L. Hong

Last updated by author(s): Mar 25, 2024

Reporting Summary

Nature Portfolio wishes to improve the reproducibility of the work that we publish. This form provides structure for consistency and transparency in reporting. For further information on Nature Portfolio policies, see our Editorial Policies and the Editorial Policy Checklist.

Statistics

For all statistical analyses, confirm that the following items are present in the figure legend, table legend, main text, or Methods section.

n/a	Confirmed	
☐	☒	The exact sample size (*n*) for each experimental group/condition, given as a discrete number and unit of measurement
☐	☒	A statement on whether measurements were taken from distinct samples or whether the same sample was measured repeatedly
☐	☒	The statistical test(s) used AND whether they are one- or two-sided<br>*Only common tests should be described solely by name; describe more complex techniques in the Methods section.*
☒	☐	A description of all covariates tested
☐	☒	A description of any assumptions or corrections, such as tests of normality and adjustment for multiple comparisons
☐	☒	A full description of the statistical parameters including central tendency (e.g. means) or other basic estimates (e.g. regression coefficient) AND variation (e.g. standard deviation) or associated estimates of uncertainty (e.g. confidence intervals)
☐	☒	For null hypothesis testing, the test statistic (e.g. *F*, *t*, *r*) with confidence intervals, effect sizes, degrees of freedom and *P* value noted<br>*Give P values as exact values whenever suitable.*
☒	☐	For Bayesian analysis, information on the choice of priors and Markov chain Monte Carlo settings
☒	☐	For hierarchical and complex designs, identification of the appropriate level for tests and full reporting of outcomes
☐	☒	Estimates of effect sizes (e.g. Cohen's *d*, Pearson's *r*), indicating how they were calculated

Our web collection on statistics for biologists contains articles on many of the points above.

Software and code

Policy information about availability of computer code

Data collection	Beckman Coulter CytExpert v2.3; Bio-Rad CFX Manager v3.1; Odyssey Infrared Imaging System v3.0.30; BioTek Gen5 v1.11.5; EnVision Manager 1.14.3049.1193
Data analysis	R v4.2.2; R Studio 2022.07.1 Build 554; tximport v1.26.1, sva (ComBat-seq) v3.46.0, DESeq2 v1.38.3, umap v0.2.10.0, ggplot v3.4.2, Hmisc v4.6-0 (R packages); Illumina Dragen v3.7.5 (WES, WGS, RNA sequencing); ichorCNA v0.10 (copy number analyses); Graphpad Prism v9.3.0; Adobe Photoshop v22.0.0; Adobe Illustrator v25.0; SyngeryFinder v3.0; FlowJo v10.8.1, fgsea_1.24.0

For manuscripts utilizing custom algorithms or software that are central to the research but not yet described in published literature, software must be made available to editors and reviewers. We strongly encourage code deposition in a community repository (e.g. GitHub). See the Nature Portfolio guidelines for submitting code & software for further information.

Data

Policy information about availability of data

All manuscripts must include a data availability statement. This statement should provide the following information, where applicable:

- Accession codes, unique identifiers, or web links for publicly available datasets
- A description of any restrictions on data availability
- For clinical datasets or third party data, please ensure that the statement adheres to our policy

Sequence data have been deposited at the European Genome-phenome Archive (EGA), which is hosted by the EBI and the CRG, under accession number EGAS00001007389. This study does not use custom code or mathematical algorithms. The uncropped immunoblotting images were exhibited in Supplementary Figure 8. The source data of the graph figures are exhibited in Supplementary Data. Plasmids herein can be found at https://www.addgene.org/Andrew_Hong/. All other data is available from the corresponding author upon reasonable request.

Field-specific reporting

Please select the one below that is the best fit for your research. If you are not sure, read the appropriate sections before making your selection.

☒ Life sciences ☐ Behavioural & social sciences ☐ Ecological, evolutionary & environmental sciences

For a reference copy of the document with all sections, see nature.com/documents/nr-reporting-summary-flat.pdf

Life sciences study design

All studies must disclose on these points even when the disclosure is negative.

Sample size	No statistical methods were used to pre-determine sample sizes for in vivo studies but our sample sizes are similar to those reported in previous publications.
Data exclusions	No data were excluded from analyses.
Replication	All experiments were performed in at least biological replicates with at least technical duplicates.
Randomization	Mice were randomized into treatment groups using Studylog software.
Blinding	For mouse studies - data collection and analysis were not performed blind to the conditions of the experiments.

Reporting for specific materials, systems and methods

We require information from authors about some types of materials, experimental systems and methods used in many studies. Here, indicate whether each material, system or method listed is relevant to your study. If you are not sure if a list item applies to your research, read the appropriate section before selecting a response.

Materials & experimental systems

n/a	Involved in the study	
☐	☒	Antibodies
☐	☒	Eukaryotic cell lines
☒	☐	Palaeontology and archaeology
☐	☒	Animals and other organisms
☐	☒	Human research participants
☒	☐	Clinical data
☒	☐	Dual use research of concern

Methods

n/a	Involved in the study	
☒	☐	ChIP-seq
☐	☒	Flow cytometry
☒	☐	MRI-based neuroimaging

Antibodies

Antibodies used	XPO1 (Santa Cruz; sc-5595), β-Actin (C-4) (Santa Cruz; sc-47778), β-Actin (Cell Signaling; 8457), TP53 (Santa Cruz; sc-126), TRIP13 (Abcam; ab128171), α-Tubulin (Santa Cruz, sc-5286), α-Tubulin (Cell Signaling; 2144), Lamin A/C (Cell Signaling; 4777 or 2032), p21 (Cell Signaling; 29475), CCND1 (Santa Cruz; sc-8396).
Validation	All antibodies are commercially available and validation information can be found at the following links: XPO1 (https://www.scbt.com/p/crm1-antibody-h-300); β-Actin (C-4) (Santa Cruz, https://www.scbt.com/p/beta-actin-antibody-c4); β-Actin (Cell Signaling, https://www.cellsignal.com/products/primary-antibodies/b-actin-d6a8-rabbit-mab/8457); TP53 (Santa Cruz, https://www.scbt.com/p/p53-antibody-do-1); TRIP13 (Abcam, https://www.abcam.com/trip13pch2-antibody-ab128171.html); α-Tubulin (Santa Cruz, https://www.scbt.com/p/alpha-tubulin-antibody-b-7); Lamin A/C (Cell Signaling, https://www.cellsignal.com/products/primary-antibodies/lamin-a-c-4c11-mouse-mab/4777 OR https://www.cellsignal.com/products/primary-antibodies/lamin-a-c-antibody/2032); p21 (Cell Signaling, https://www.cellsignal.com/products/primary-antibodies/p21-waf1-cip1-12d1-rabbit-mab/2947); CCND1 (Santa Cruz, https://www.scbt.com/p/cyclin-d1-antibody-a-12).

Eukaryotic cell lines

Policy information about cell lines

Cell line source(s)	All cell lines were established from patient-derived tumor samples or PDX tumor samples.
Authentication	Whole genome sequencing (WGS), whole exome sequencing (WES), and RNA sequencing were performed on all samples.
Mycoplasma contamination	Cell lines were tested for mycoplasma contamination using the Lonza Mycoplasma kit.

Commonly misidentified lines (See ICLAC register)	No commonly misidentified cell lines were used in this study.

Animals and other organisms

Policy information about studies involving animals; ARRIVE guidelines recommended for reporting animal research

Laboratory animals	Female NSG (NOD-scid IL2Rgamma null) mice, 6-weeks old.
Wild animals	This study did not involve wild animals.
Field-collected samples	This study did not involve field-collected samples.
Ethics oversight	The study was conducted at Dana-Farber Cancer Institute (DFCI) in an AAALAC accredited vivarium with the approval of the Institutional Animal Care and Use Committee.

Note that full information on the approval of the study protocol must also be provided in the manuscript.

Human research participants

Policy information about studies involving human research participants

Population characteristics	Parents of children with a new renal mass (and if of age of assent, the child as well) were consented (and for child at age of assent, assented) to enroll on IRB approved biology protocols at each institution. Samples with a pathological diagnosis of Wilms Tumor were then used in this study.
Recruitment	No bias with regards to ethnicity, race and or gender.
Ethics oversight	Samples were obtained under protocols approved by the IRB at the Dana Farber Cancer Institute/Boston Children's Cancer and Blood Disorders Center OR the IRB at the Aflac Cancer and Blood Disorders Center at the Children's Healthcare of Atlanta and Emory University School of Medicine.

Note that full information on the approval of the study protocol must also be provided in the manuscript.

Flow Cytometry

Plots

Confirm that:

- [x] The axis labels state the marker and fluorochrome used (e.g. CD4-FITC).
- [x] The axis scales are clearly visible. Include numbers along axes only for bottom left plot of group (a 'group' is an analysis of identical markers).
- [x] All plots are contour plots with outliers or pseudocolor plots.
- [x] A numerical value for number of cells or percentage (with statistics) is provided.

Methodology

Sample preparation	Cell lines were established from patient samples. Cells were treated with drug or control as described in Methods. Cells were harvested and washed twice with PBS. Cells were fixed in 80% ethanol for two hours before staining with propidium iodide.
Instrument	Beckman Coulter Cytoflex Model No. B75442
Software	Data was collected with Beckman Coulter CytExpert v2.3. Data was analyzed with FlowJo v10.8.1.
Cell population abundance	50,000 events were recorded for all samples. Singlets were gated and identified as described below.
Gating strategy	Debris was removed via gating under FSC-A/SSC-A. Singlets were gated with FSC-A/FSC-H. Cell cycle phases were determined using the Watson (Pragmatic) model in FlowJo v10.8.1.

- [x] Tick this box to confirm that a figure exemplifying the gating strategy is provided in the Supplementary Information.
